# The Role of MicroRNAs in Early Chondrogenesis of Human Induced Pluripotent Stem Cells (hiPSCs)

**DOI:** 10.3390/ijms20184371

**Published:** 2019-09-05

**Authors:** Ewelina Stelcer, Katarzyna Kulcenty, Marcin Rucinski, Karol Jopek, Magdalena Richter, Tomasz Trzeciak, Wiktoria Maria Suchorska

**Affiliations:** 1 Radiobiology Lab, Greater Poland Cancer Centre, Garbary 15th Street, 61-866 Poznan, Poland (K.K.) (W.M.S.); 2 Department of Histology and Embryology, Poznan University of Medical Sciences, Swiecickiego 6 Street, 60-781 Poznan, Poland (M.R.) (K.J.); 3 Department of Electroradiology, Poznan University of Medical Sciences, Garbary 15th, 61-866 Poznan, Poland; 4 Department of Orthopedics and Traumatology, Poznan University of Medical Sciences, 18 czerwca 1956r Street, 61-545 Poznan, Poland (M.R.) (T.T.); 5 Center for Advanced Technology, Adam Mickiewicz University, Uniwersytetu Poznańskiego 10, 61-614 Poznan, Poland

**Keywords:** human induced pluripotent stem cells, differentiation, chondrogenesis, microRNAs, microarrays

## Abstract

Human induced pluripotent stem cells (hiPSCs) play an important role in research regarding regenerative medicine. Particularly, chondrocytes differentiated from hiPSCs seems to be a promising solution for patients suffering from osteoarthritis. We decided to perform chondrogenesis in a three-week monolayer culture. Based on transcriptome analysis, hiPSC-derived chondrocytes (ChiPS) demonstrate the gene expression profile of cells from early chondrogenesis. Chondrogenic progenitors obtained by our group are characterized by significantly high expression of Hox genes, strongly upregulated during limb formation and morphogenesis. There are scanty literature data concerning the role of microRNAs in early chondrogenesis, especially in chondrogenic differentiation of hiPSCs. The main aim of this study was to investigate the microRNA expression profile and to select microRNAs (miRNAs) taking part in early chondrogenesis. Our findings allowed for selection crucial miRNAs engaged in both diminishing pluripotency state and chondrogenic process (inter alia hsa-miR-525-5p, hsa-miR-520c-3p, hsa-miR-628-3p, hsa-miR-196b-star, hsa-miR-629-star, hsa-miR-517b, has-miR-187). These miRNAs regulate early chondrogenic genes such as: *HOXD10*, *HOXA11*, *RARB*, *SEMA3C*. These results were confirmed by RT-qPCR analysis. This work contributes to a better understanding of the role of miRNAs directly involved in chondrogenic differentiation of hiPSCs. These data may result in the establishment of a more efficient protocol of obtaining chondrocyte-like cells from hiPSCs.

## 1. Introduction

Human induced pluripotent stem cells (hiPSCs), reprogrammed from fully differentiated cells such as fibroblasts, play an important role in research regarding modern regenerative medicine [1]. They constitute a very promising approach because of their unique properties: Unlimited self-renewal capacity and potential to differentiate into derivatives of three primary germ layers (ecto-, endo-, and mesoderm). Consequently, they can give rise to new types of cells such as cardiomyocytes, pancreatic β cells, neurons, chondrocytes, and osteocytes [2]. Particularly, chondrocytes differentiated from hiPSCs seems to be a very interesting and alternative solution for patients suffering from currently unresponsive disorders such as osteoarthritis [3]. Obtaining this type of cells from hiPSCs is widely described in the literature (e.g., differentiation in vitro in monolayer culture, through pellets, via embryoid bodies (EBs) as well as in 3D culture using the wide variety of bioscaffolds) [4,5]. We decided to perform chondrogenesis in a three-week monolayer culture due to the fact that this approach is cost and time effective. The advantage of this directed differentiation protocol is that, unlike most other differentiation techniques (such as EB formation), our protocol requires no additional and time-consuming steps. It involves the use of chondrogenic medium with the addition of selected growth factors (GFs) such as transforming growth factor beta 3 (TGF-β3), bone morphogenetic protein 4 (BMP-4), and insulin-like growth factor 1 (IGF-1) [6]. In our previous studies, we demonstrated that these hiPSC-derived chondrocytes (ChiPS cells) possess properties of chondrogenic progenitors. They reveal presence of type II collagen, cartilage oligomeric matrix protein (COMP), and CD44; however, based on transcriptome analysis they mainly demonstrate the gene expression profile of cells from early chondrogenesis [7]. The chondrogenic progenitors obtained by our group are characterized by significantly high expression of Hox genes (*HOXD13*, *HOXB6*, *HOXC11*, *HOXA6*, *HOXA11*, and *HOXD10*) strongly upregulated during limb formation and limb morphogenesis. It is possible that those cells will undergo terminally spontaneous chondrogenic differentiation and/or also have a stimulating effect on other cells present in the microenvironment after implementation to the joint [8]. Apart from that, we demonstrated that during chondrogenic differentiation, in vitro signaling pathways involving p53 and DNA damage response are notable upregulated [9].

MicroRNAs (miRNAs) are small non-coding RNAs with a length of about 21–22 nucleotides that control many physiological processes such as apoptosis, cell cycle, angiogenesis, and aging [10]. They also play an important role in reprogramming, pluripotency maintenance, proliferation, and differentiation of stem cells (SCs); as additionally, they are actively engaged in the pathogenesis of many diseases and different signaling pathways [11,12,13]. Their function is based on the ability to inhibit translation or promote the degradation of mRNA [14]. There are scanty literature data concerning the role of microRNAs in early chondrogenesis, especially in chondrogenic differentiation of hiPSCs. 

**Figure 1:**
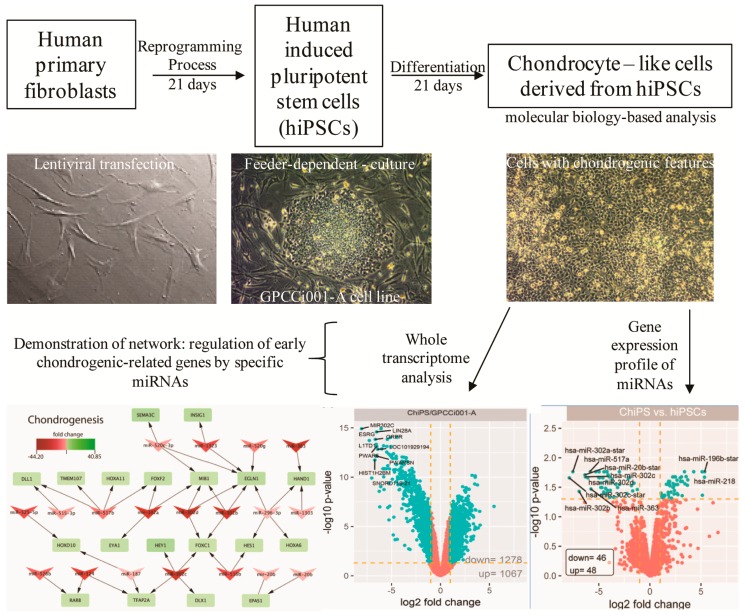
Scheme of experiment. The primary human dermal fibroblasts (image: 20× magnification) from a breast cancer patient were obtained to generate human induced pluripotent stem cells (hiPSCs: GPCCi001-A cell line) (image: 10× magnification) by the use of lentiviral transfection [15]. Then, hiPSCs underwent chondrogenic differentiation via monolayer culture with the addition of growth factors for 21 days. The obtained chondrocyte-like cells (image: 10x magnification) demonstrate the presence of markers specific for human chondrocytes (inter alia: The presence of type II collagen) [6]. To take a closer look at obtained chondrocyte-like cells derived from hiPSCs, their gene expression profile was investigated. It was revealed that these cells activate signaling pathways characteristic of cells from early stages of chondrogenesis [7,9,16]. In this study, we aimed to investigate the relationship between microRNAs (miRNAs) and most highly expressed early chondrogenic genes regulated by them. Interestingly, we indicated several new miRNAs engaged in chondrogenesis of hiPSCs.

In this context, the main aim of this study was to investigate the microRNA expression profile based on the Affymetrix platform (1) and using microarrays to select key miRNAs taking part in early chondrogenesis (2) and in attenuation of the pluripotency state (3) (Figure 1).

Our findings allowed for the selection of crucial miRNAs engaged in both diminishing the pluripotency state (inter alia hsa-miR-302a-5p, hsa-miR-302c-5p, hsa-miR-302b, hsa-miR-302d) and chondrogenic process (inter alia hsa-miR-525-5p, hsa-miR-520c-3, hsa-miR-628-3p, hsa-miR-196b-star, hsa-miR-629-star, hsa-miR-517b, has-miR-187). These miRNAs regulate early chondrogenic genes such as: *HOXD10*, *HOXA11*, *RARB*, *SEMA3C*. These results were appropriate visualized and confirmed by RT-qPCR analysis (hsa-miR-302a-star, hsa-miR-302c-star, hsa-miR-525-5p, hsa-miR-520c-3p, hsa-miR-628-3p, hsa-miR-196b-star, hsa-miR-629-star). 

This work contributes to a better understanding of the role of miRNAs directly involved in chondrogenic differentiation of hiPSCs. It is likely that this is the first report concerning selection of key miRNAs responsible for regulating the first stages of chondrogenesis. These data may result in the establishment of a more efficient protocol of obtaining chondrocyte-like cells from hiPSCs.

## 2. Results

### 2.1. Chondrogenic Differentiation of hiPSCs: We Obtained Chondrogenic Progenitors from hiPSCs

We applied a chondrogenic protocol that was previously published [6]. The results indicate that we obtained chondrocyte-like cells (ChiPS) expressing markers characteristic for mature chondrocytes (e.g., aggrecan) (Supplementary Figure S1), moreover, we demonstrated that ChiPS reveal the gene expression profile of cells from early chondrogenesis (e.g., *HOX* genes) [7].

### 2.2. Analysis of Microarray Experiments of miRNA Expression Profiling: ChiPS and hiPSCs Are Characterized by Distinct miRNA Expression Profiles

**Figure 2:**
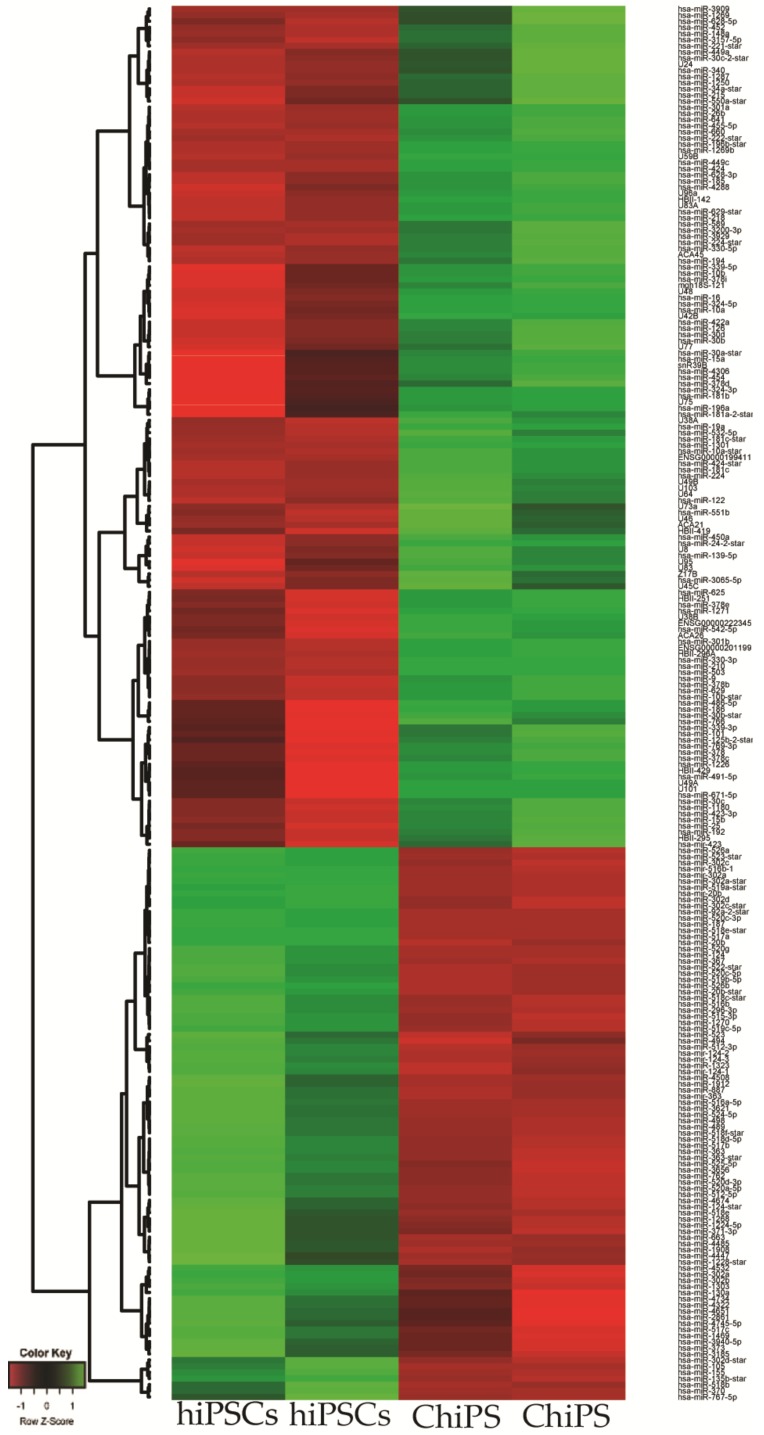
Heatmap graphs of the miRNAs in the experimental groups: chondrocytes derived from hiPSCs (ChiPS) vs. hiPSC. Arbitrary signal intensity acquired from the microarray analysis is represented by the colors (green = higher expression; red = lower expression). Log2 signal intensity values for any single gene were resized to row Z-score scales.

A GeneChip™ miRNA 3.1 Array Strip (Thermo Fisher Scientific) was used to analyze and compare miRNA expression profiles of chondrocytes derived from hiPSCs (ChiPS) and hiPSCs (GPCCi001-A cell line). The miRNA expression profile of ChiPS significantly differs from that obtained from hiPSCs. The significantly different miRNAs were subject to hierarchical clustering and are shown on heatmap (Figure 2). 

**Figure 3:**
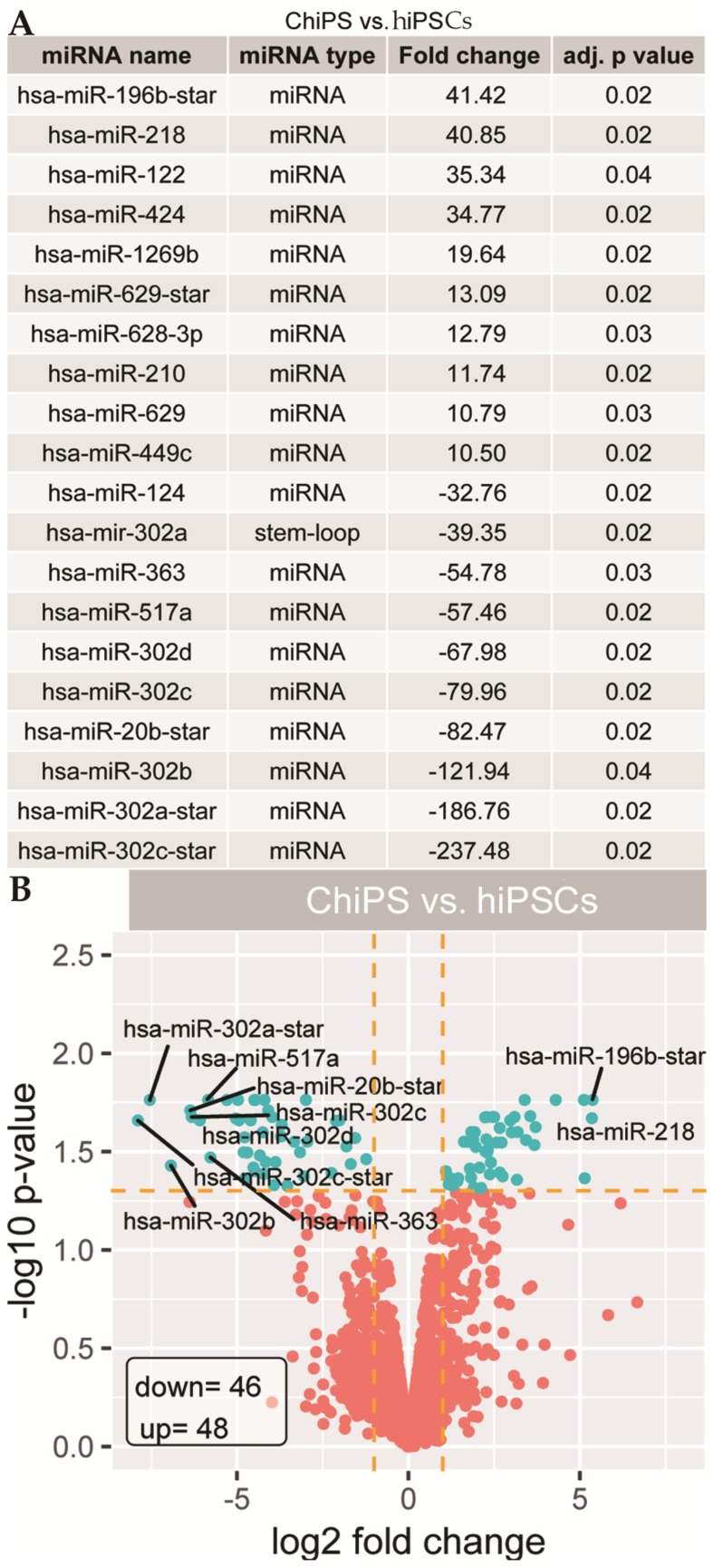
The table shows 20 miRNAs with the highest (10 miRNAs) and lowest (10 miRNAs) fold changes from the lists of differentially expressed miRNAs between ChiPS and hiPSCs (**A**). The volcano plot shows the total miRNAs expression profiles of the ChiPS and hiPSC experimental groups. Each dot represents the mean expression level of a single miRNA obtained from a microarray normalized dataset. The orange dotted lines (cut-off values) were established according to the following parameters: |fold| > 2 and adjusted (adj.) *p*-value < 0.05. miRNAs above the cut-off are considered to be differentially expressed and are shown as blue dots. The total number of differentially expressed miRNAs are displayed in the bottom left corner of the graph (**B**).

The adopted cut-off criteria were |FC| > 2 and *p*-value < 0.05. The top 20 highly differentially expressed miRNAs were characterized by high fold change (FC) values ranged for upregulated miRNAs from 10.5 to 41.42 and for downregulated genes from −32.76 to −247.48 (Figure 3A). On the basis of analysis of top 10 downregulated miRNAs in ChiPS, we found those belonging to the miR-302 family (hsa-miR-302a: −39.35; hsa-miR-302d: −67.98; hsa-miR-302c: −79.96; hsa-miR-302b: −121.94; hsa-miR-302a-star; −186.76; hsa-miR-302c-star: −237.48), clustered miRNAs (has-miR-363: −57.46; has-miR-20b-star: −82.47) as well as hsa-miR-124 (−32.76) and has-miR-517a (−57.46). We also demonstrated the top 10 upregulated miRNAs in ChiPS: hsa-mir-129b-star (41.42), hsa-mir-218 (40.85), hsa-mir-122 (35.34), hsa-mir-424 (34.77), hsa-mir-1269b (19.64), hsa-mir-629-star (13.09), hsa-mir-628-3p (12.79), hsa-mir-210 (11.74), hsa-mir-629 (10.79), hsa-mir-449c (10.5). In general, we found 46 downregulated miRNAs and 48 upregulated miRNAs that met cut-off criteria in ChiPS versus hiPS cells; those results were visualized as a volcano plot (Figure 3B).

### 2.3. Biological Processes Regulated by miRNAs during Chondrogenic Differentiation: miRNAs Strongly Regulate Signaling Pathways Engaged in Early Chondrogenesis

We used DAVID (Database for Annotation, Visualization, and Integrated Discovery) to verify the processes regulated by significantly differentially expressed miRNAs. Firstly, targets for miRNAs were selected based on the SpidermiR package where target genes were predicted by screening of five miRNA-target gene databases (DIANA, Miranda, PicTar, TargetScan, miRTar, miRWalk).

We found, predicted, and validated target genes for our 94 differentially expressed miRNAs. The level of expression of target genes was determined using our previously published data from the Gene Expression Omnibus (GEO) database (accession number: GSE108035) [7].

**Figure 4:**
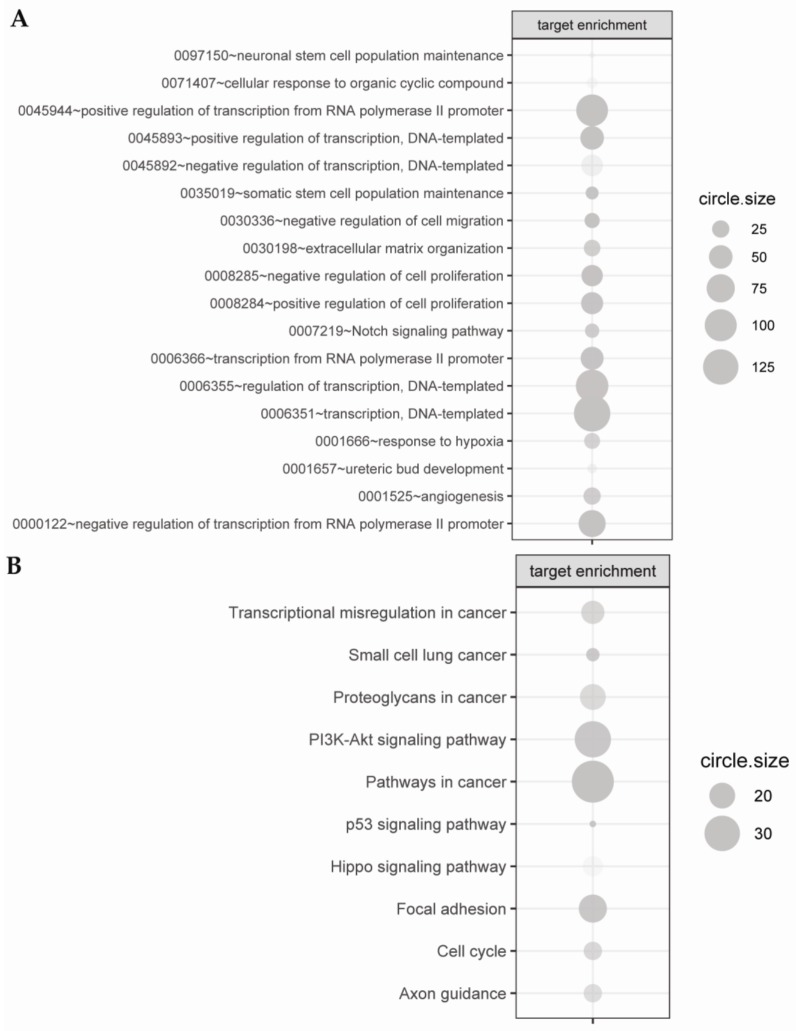
miRNA targets were assigned to Gene Ontology—Biological Process (GO.BP) (**A**) and Kyoto Encyclopedia Genes and Genomes (KEGG) databases (**B**). The size of grey circles represents the number of target genes regulating the described signaling pathways. The intensity of grey color symbolizes the statistical significance (the darker the shade of green, the lower adj. *p*-value).

Based on the assumption that elevated expression of miRNA expression results in decreased expression of target gene (and vice versa), for subsequent analysis, we selected those target genes that were above the cut-off criteria (|fold| > 2 and *p* < 0.05) in which the FCs were inversely correlated with the FCs of miRNAs. These criteria were fulfilled by only 803 unique target genes. miRNA targets were then assigned to Gene Ontology (GO) (Figure 4A) and Kyoto Encyclopedia Genes and Genomes (KEGG) (Figure 4B) databases.

According to GO analysis, during chondrogenic differentiation of hiPSCs, there are significantly differentially expressed miRNA-target pairs, playing an important role in the regulation of signaling pathways, inter alia: “response to hypoxia (0001666)”, “negative regulation of cell migration (0030336)”, “Notch signaling pathway (0007219)”, “angiogenesis (0001525)”, “extracellular matrix organization (0030198)”. Other processes: “transcriptional misregulation in cancer”, “small cell lung cancer”, “proteoglycans in cancer”, “PI3K-Akt signaling pathway”, “pathways in cancer”, “p53 signaling pathway”, “Hippo signaling pathway”, “focal adhesion”, “cell cycle”, and “axon guidance” were revealed using the KEGG database.

**Figure 5:**
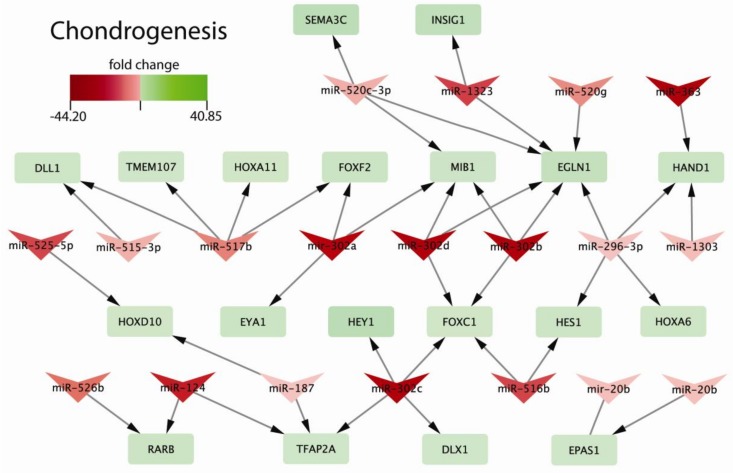
The relationship between miRNAs and the genes regulated by them engaged in early chondrogenesis. ChiPS cells demonstrate markers for chondrogenic progenitors and upregulated expression of genes of early chondrogenesis (green color). Those genes are regulated by miRNAs whose expression decreases during chondrogenic differentiation (red color). The intensity of the used color of miRNAs and regulated genes was based on their fold change (FC) values (ChiPS vs. hiPSCs). miRNAs regulate sets of genes at the same time.

We found, that early chondrogenic-related genes were controlled by the range of miRNAs: *SEMA3C* (miR-520c-3p), *INSIG1* (miR-1323), *DLL1* (miR-515-3p, miR-517b), *TMEM107* (miR-517b), *HOXA11* (miR-517b), *FOXF2* (miR-517b, miR-302a), *MIB1* (miR-302a, miR-302d, miR-302b), *EGLN1* (miR-302d, miR-302b, miR-296-3p), *HAND1* (miR-296-3p, miR-1303), *HOXD10* (miR-525-5p, miR-187), *EYA1* (miR-302a), *HEY1* (miR-302c), *FOXC1* (miR-302c, miR-516b), *HES1* (miR-296-3p, miR-516b), *HOXA6* (miR-296-3p), *RARB* (miR-526b, miR-124), *TFAP2A* (miR-124, miR-187, miR-302c), DLX1 (miR-302c), and EPAS1 (miR-20b) (Figure 5). These genes are engaged in the processes taking place during limb formation (embryonic limb morphogenesis: *RARB*, *HOXA11*, *HOXD10, SEMA3C, TMEM107;* limb development: *SEMA3C*, *RARB*, *HOXA11*, *HOXD10*, *TMEM107*, *TFAP2A*; embryonic skeletal system development: *EYA1*, *HOXA6*, *DLX1*, *HOXA11*, *HOXD10*, *TFAP2A*; embryonic skeletal system morphogenesis: *EYA1*, *HOXA6*, *HOXA11*, *HOXD10*, *TFAP2A;* embryonic organ development: *HEY1*, *INSIG1*, *EGLN1*, *HAND1*, *FOXF2*, *EYA1*, *RARB*, *HES1*, *DLL1*, *HOXA6*, *FOXC1*, *HOXA11*, *HOXD10*, *EPAS1*, *TFAP2A*; embryonic organ morphogenesis: *INSIG1*, *HAND1, FOXF2, EYA1, RARB, HES1, DLL1, HOXA6, FOXC11, HOXA11, HOXD10, TFAP2A).*

**Figure 6:**
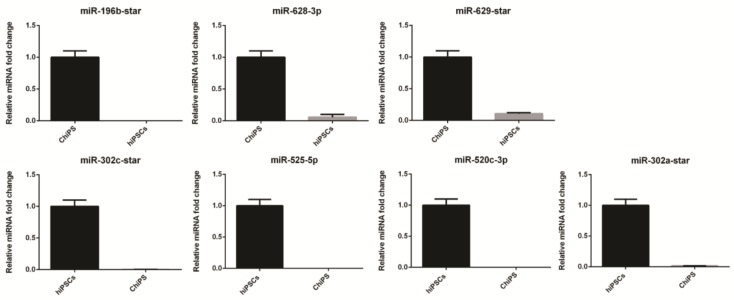
Real time qPCR validation of microarray data. For validation, we selected the most up- and downregulated miRNAs according to the previously created table (Figure 3) and Figure 5 and relationship between miRNAs and the genes regulated by them engaged in early chondrogenesis (Figure 4). The panel represents normalized ChiPS/hiPSCs fold changes (FCs) of selected miRNAs based on microarray data.

The most up- and downregulated miRNAs were verified by the RT-qPCR technique: hsa-miR-196b-star, hsa-miR-628-3p hsa-miR-629-star, hsa-miR-302c-star, hsa-miR-525-5p, hsa-miR-520c-3p, and hsa-miR-302a-star (Figure 6).

## 3. Discussion

Barter et al. demonstrated that miR-140 (mainly the -5p strand) and miR-455 are essential for chondrogenic differentiation of mesenchymal stem cells (MSCs). They highlighted the relevant role of miR-140-5p in the regulation of—important for chondrogenesis and skeletal development—Wnt signaling pathway. This signaling pathway is responsible for cell cycle progression and cell proliferation, as additionally, it also contributes to promoting MSC and chondrocyte proliferation via miR-140 [17]. Mahboudi and colleagues (2018) made a step further toward the improvement of chondrogenesis because they differentiated hiPSCs by taking advantage of the overexpression of miR-140 and the use of TGF-β3 in a high-cell density culture. Those cells revealed a higher level of SRY (Sex determining region Y)- box gene 9 (SOX9), type II collagen, and aggrecan in comparison with traditional method of MSC differentiation [18]. However, it is important to point out that MSCs tend to simultaneously acquire hypertrophic properties during chondrogenic induction, indicating the possibility of further differentiation toward endochondral bone formation [19]. On the contrary, we observed the decreased expression of members of Wnt signaling pathway (Supplementary Figure S2). It may be explained by the fact that, as we mentioned previously, we obtained chondrogenic progenitors rather than mature chondrocytes. However, on the other hand, it may also indicate that our cells lost their pluripotent nature since it is well known that Wnt signaling pathways play an important role in maintaining the pluripotency state in human embryonic stem cells and hiPSCs. Thus, it is likely that ChiPS undergo the first stages of chondrogenesis [20,21].

Interestingly, Chen and co-workers revealed that miRNA-218 takes part in promoting early chondrogenesis of MSCs with simultaneous inhibition of later chondrocyte maturation. It is also connected with elevated expression of chondrogenic markers: *SOX9*, *COL2A1*, *ACAN*, *GAG,* and *COMP* and decreased level of osteogenic markers: *COL10A1*, *MMP-13,* and *VEGF*. Those authors suggested that miR-218 regulates functioning of 15-hydroxyprostaglandin dehydrogenase (HPGD), which is very important for effective chondrogenic differentiation [22]. It is a very optimistic report because we also demonstrated the elevated expression of miR-218 (FC = 40.85 in ChiPS vs. hiPSCs).

The available evidence indicates that miR-145 regulates chondrogenic differentiation of MSCs by targeting *SOX9*. Overexpression of miR-145 leads to the diminished mRNA levels of chondrogenic markers (collagen type II alpha 1 chain (COL2A1), aggrecan, COMP, (collagen type IX alpha 2 chain (COL9A2), and collagen type XI alpha 1 chain (COL11A1)) in C3H10T1/2 cells. Thus, miR-145 is a negative regulator of early chondrogenesis [23]. In our study, we did not observe the statistically significant change of expression of miR-145.

Gao and collaborators (2018) investigated the influence of miR-101 on chondrogenic differentiation of rat bone MSCs (BMSCs). They proved that miR-101 is upregulated during rat BMSC chondrogenic differentiation. It is accompanied by the elevated level of *SOX9* and decreased level of *RUNX2*. Therefore, miR-101 may constitute a novel therapeutic target for the treatment of osteoarthritis (OA) [24]. Those results also correspond with our data: We also obtained the elevated level of miR-101 expression (FC = 2.57); however, this value was not statistically significant (adj. *p* = 0.15) (Supplementary Table S1).

According to Guerit et al. the downregulation of miR-29a and upregulation of its target *FOXO3A*, is essential for the differentiation of MSCs into chondrocytes and for in vivo cartilage formation. The authors showed that Akt-Forkhead box O (Akt-FoxO) pathways enhanced chondrocyte proliferation but also inhibited chondrocyte maturation and matrix production [25]. In our previous study [7], we observed the increased level of another member of the human Forkhead box (Fox gene family)—*FOXF2* [26], which may confirm the important role of this family in early chondrogenesis.

Another interesting study [27], involves the evaluation of the role of miRNAs in human adipose-derived SCs (hADSCs) chondrogenesis. The following upregulated miRNAs can be determined: miR-193b, miR-199a-3p/hsa-miR-199b-3p, miR-455-3p, miR-210, miR-381, miR-92a, miR-320c, and miR-136. Our study also proved that miR-210 is significantly upregulated (FC = 11.74). Apart from that, we revealed the elevated level of miRNA belonging (as mentioned by the authors above, mir-455-3p) to the miR-455 family: miR-455-5p-star, however without achieving statistical significance: (FC = 2.33; adj. *p* = 0.04). Our findings have demonstrated an extremely high level of miR-196b-star (FC = 41.42). Based on literature data, miRNAs-196 are expressed from *HOX* gene clusters (groups of related transcription factor genes crucial for numerous developmental program such as limb development and embryonic skeletal system development). Moreover, these gene clusters are targets of miR-196 [28]. Consequently, the high expression of miR-196b-star (FC = 41.42), demonstrated by our group, is in parallel with high expression of genes belonging to HOX gene clusters: *HOXD13*, *HOXB6*, *HOXC11*, *HOXC9*, *HOXA6*, *HOXA11*, and *HOXD10* as we demonstrated previously [7]. Another study confirms that miR-196b expression patterns indicate a potential role in regulating specific phases of chondrogenesis. Its higher levels were found to be higher in isolated precursors or differentiated in comparison to hypertrophic chondrocytes derived from human frozen blocks of human embryonic limb tissues. Apart from that, miR-196 sub-types (including both miR-196a and miR-196b, which are almost identical in sequence but located on different chromosomes) have been reported to regulate skeletal patterning in zebrafish, chicken, and salamander [29].

It is important to point out that we also revealed high expression of miR-122 (FC = 35.34) and miR-424 (FC = 34.77). Literature data indicate that both these miRNAs are probably responsible for the normal course of chondrogenesis. The decrease of miR-122-3p is characteristic for MSCS derived from orthopedic disease—steroid-induced osteonecrosis of the femoral head (SONFH) [30]—and miR-122-5p for serum from patients suffering from osteoporosis [31]. In turn, miR-424 is considered as anti-angiogenic miRNAs because of its capacity to repress vascular endothelial growth factor receptor 2 (VEGFR2), fibroblasts growth factor receptor 1 (FGFR1), and vascular endothelial growth factor (VEGF) what is characteristic for early steps of chondrogenesis [32].

We also demonstrated upregulation of miR-628-3p (FC = 12.79) which was subsequently confirmed by the use of RT-qPCR technique. miR-628-5p was significantly upregulated in exosomal patients from MSCs induced to undergo chondrogenesis, which highlights its important role in the regulation of chondrogenesis [33]. A very nice summary of miRNAs taking part in the regulation of MSCs chondrogenesis was done by Li et al. (2017). They described the role of the most important chondrogenesis-related miRNAs: miR-138, miR-23b, and miR-335-5p [10].

However, it is important to emphasize that, all above-described literature data concerns MSCs and data concerning the role of miRNAs in chondrogenic differentiation of pluripotent SCs, particularly hiPSCs, are lacking. This is the first report involving miRNA expression profile of hiPSCs undergoing early chondrogenesis.

## 4. Materials and Methods

### 4.1. Differentiation of hiPSCs into Chondrogenic Progenitors

In this experiment, we used the feeder-dependent hiPSCs cell line (GPCCi001-A) which was created by our group [15] from fibroblasts in the reprogramming process. In the next step, hiPSCs were differentiated into ChiPS via three-week monolayer culture in chondrogenic medium (with the addition of L-proline, ascorbic acid, sodium pyruvate, insulin–transferrin–selenium (ITS)  +  Premix, and dexamethasone) supplemented with GFs (basic fibroblast growth factor (FGF-2); BMP-4; platelet-derived growth factor (PDGF-BB); TGF-β3, and IGF-1) (Supplementary Figure S1) [6]. The obtained chondrogenic progenitors were analyzed using the following techniques: immunofluorescence (e.g., COMP, type II collagen), flow cytometry (e.g., CD44 and CD151), and qPCR (SOX-trio genes). We also performed whole-transcriptome analysis through microarrays [7,9,16].

### 4.2. Immunofluorescence Analysis

The cells were transferred into a 0.1% gelatin-coated 48-well plate for 48 h, and then washed with phosphate buffered saline (PBS) (Sigma Aldrich, Saint Louis, MO, USA) and fixed for 20 min in 4% formaldehyde (CHEMPUR, Piekary Slaskie, Poland) (400 μL of formaldehyde per well). Then, the cells were rinsed with PBS containing 1% bovine serum albumin (BSA) (Sigma Aldrich, Saint Louis, MO, USA) and incubated for 30 min in PBS containing 1% BSA and 0.2% Triton X-100 (Sigma Aldrich, Saint Louis, MO, USA). After 30 min, the cells were washed with PBS containing 1% BSA. The primary antibodies were diluted in PBS containing 1% BSA and 0.2% Triton X-100, and the cells were incubated overnight at 4 °C with the following primary antibodies (all from Abcam PLC, Cambridge, UK): type II collagen (1:100) (ab34712); type IX collagen (1:100) (ab134568); aggrecan (1:85) (ab3778); COMP (1:100) (ab128893). After conjugation with the primary antibodies, the cells were rinsed three times with PBS containing 1% BSA. The following secondary antibody was diluted with 1% BSA in PBS and incubated in the dark for 1 h at 37 °C: Rabbit polyclonal antibody (1:500) (711-546-152) and mouse monoclonal antibody (1:500) (711-545-150) (both from Jackson ImmunoResearch, Philadelphia, PA, USA). After the cells were washed three times with 1% BSA in PBS, they were stained for 5 min with diamidino-2-phenylindole dye (DAPI) (Sigma Aldrich, Saint Louis, MO, USA) solution in water (1:10,000) and then washed with PBS before undergoing microscope analysis.

### 4.3. Total RNA and miRNA-Enriched Fraction Purification

Total RNA and miRNA-enriched fraction from hiPSCs and hiPSCs after chondrogenic differentiation (ChiPS) were purified with miRNeasy Kit and RNeasy MinElute according to the manufacturer’s instructions (217004, Quiagen, Hilden, Germany).

### 4.4. Gene Expression Profile of miRNAs

The profiling of miRNA expression was conducted using Affymetrix platform-based microarrays with GeneChip™ miRNA 3.1 Array Strip (Thermo Fisher Scientific, Waltham, MA, USA). Each microarray was designed according to miRBase Release 17 database that contained complementary probes to detect: 1733 human mature miRNA, 2216 human snoRNA, CDBox RNA, H/ACA Box RNA, and scaRNA, 1658 human pre-miRNA. The procedure of miRNA hybridization was performed using the FlashTagTM Biotin HSR RNA Labeling Kit (Thermo Fisher Scientific, Waltham, MA, USA). Briefly, 150 ng of previously isolated miRNA was subjected to poly(A) tailing and biotin ligation according to the producer’s protocol. Biotin-labeled miRNA was hybridized to GeneChip™ miRNA 3.1 Array Strip (20 h, 48 °C). Then, the microarrays were washed and stained applying the Affymetrix GeneAtlas Fluidics Station (Affymetrix, Santa Clara, CA, USA) according to the manufacturer’s instructions. Then, the array strips were scanned using an Imaging Station of GeneAtlas System (Thermo Fisher Scientific, Waltham, MA, USA). The preliminary analysis of scanned chips was carried out with the use of Affymetrix GeneAtlas Operating Software (Affymetrix, Santa Clara, CA, USA). The quality of the gene expression data was verified using the quality control criteria established by the software.

The obtained CEL files were imported into downstream data analysis using BioConductor package with relevant BioConductor libraries from statistical R programing language. The Robust Multiarray Average (RMA) normalization algorithm implemented in the “Affy” library was used for normalization, background correction, and calculation of the expression values of all of the examined miRNAs [34]. Biological annotation was obtained from the pd.mirna.3.1 library where annotated data frame object was merged with normalized data set, leading to a complete miRNA data table. Differential expression and statistical assessment were determined by applying the linear model for microarray data implemented in the “limma” library [35]. The selection criteria of a significantly changed gene expression were based on fold difference higher than absolute 2 and p-value after false discovery rate (FDR) correction < 0.05. Differentially expressed miRNA were also subjected to a hierarchical clusterization algorithm and visualized as a heat map. The result was presented as volcano plot, showing the total number of up- and downregulated miRNAs.

### 4.5. miRNA-Target Gene Prediction

To identify potential target genes for differentially expressed miRNA, we took advantage of a SpidermiR package. Differentially expressed miRNAs were used as a query for searching target genes in the following databases: For predicted targets—DIANA, Miranda, PicTar, TargetScan, and for experimentally validated targets—miRTar, miRWalk [36]. To determine the actual expression value of selected target genes, we have used our data previously deposited in the Gene Expression Omnibus (GEO) database (accession number: GSE108035) [7]. Obtained FC values for mRNA were assigned to the target genes’ data table. Subsequently, we selected only those target genes for which FC was inversely correlated with the FC value of appropriate miRNA (cutoff criteria: fold ± 2, adj. *p*-value < 0.05). This selected set of unique target genes was subjected to functional annotation and clusterization using the DAVID (Database for Annotation, Visualization, and Integrated Discovery) bioinformatics tool [37,38]. Gene symbols of differentially expressed genes were uploaded to DAVID by the “RDAVIDWebService” BioConductor library [39], where DEGs were assigned to relevant Gene Ontology (GO) terms, with subsequent selection of significantly enriched GO terms from GO BP Direct and KEGG (Kyoto Encyclopedia Genes and Genomes) database. The *p*-values of selected GO terms were corrected using Benjamini–Hochberg correction described as adjusted *p*-values [40]. Relevant GO ontological groups with adjusted *p*-values below 0.05 and N per group > 5 were visualized using bubble plot. Interactions between miRNA and target genes, in selected GO BP or KEGG terms, were visualized using Cytoscape 3.7.1 [41].

### 4.6. Validation of miRNAs Expression

To validate differentially expressed microRNA selected in microarray expression profiling microRNA from hiPSCs and hiPSC-derived chondrogenic progenitors were reverse transcribed with TaqMan™ MicroRNA Reverse Transcription Kit (Applied Biosystems, Foster City, CA, USA). Quantitative real-time PCR analysis was performed with TaqMan miRNA Assay specific to chosen miRNA and TaqMan™ Universal PCR Master Mix (Applied Biosystems, Foster City, CA, USA). The internal control U6 snRNA (RNU6B; Applied Biosystems) served as an endogenous control and was utilized for RT-qPCR result normalization.

All real-time based analyses were performed on a Cobas Z480 device with LightCycler 480 Software (Roche, Basel, Switzerland). Statistical analyses of obtained results were conducted in GraphPad Prism 6 (GraphPad Software, San Diego, CA, USA), using Student’s *t*-test.

## 5. Conclusions

In this paper, we have underlined the superior role of miRNAs in regulating signaling pathways engaged in early chondrogenesis (e.g., limb development, embryonic limb morphogenesis, and embryonic skeletal system development). We have also demonstrated that during early chondrogenesis in vitro miRNAs play a crucial role in controlling chondrogenic-related genes. Based on miRNA expression profiling we have selected crucial miRNAs taking part in those processes: hsa-miR-196b-star and has-miR-629-star and hsa-miR-628-3p. In the near future, this knowledge may be useful within the gene therapy domain. Consequently, some miRNAs described in this paper may serve as potential target for further improvement of obtaining chondrocyte-like cells throughout using knock-down and overexpression techniques. It may result in increase of efficiency of chondrogenesis or decrease of pluripotency state during differentiation. According to our knowledge, it is the first report regarding the role of miRNAs in early chondrogenesis of hiPSCs, thus, more data based on small RNA molecules are needed to better understand key aspects of the nature of chondrogenic hiPSC differentiation.

## Supplementary Materials

Supplementary materials can be found at https://www.mdpi.com/1422-0067/20/18/4371/s1.

## Abbreviations

ACAN	Aggrecan
BMP-4	Bone morphogenetic protein 4
bMSCs	Bone mesenchymal stem cells
COL2A1	Collagen type II alpha 1 chain
COL10A1	Collagen type X alpha 1 chain
COL11A1	Collagen type XI alpha 1 chain
COMP	Cartilage oligomeric matrix protein
DAVID	Database for Annotation, Visualization, and Integrated Discovery
EBs	Embryoid bodies
FGF-2	Basic fibroblast growth factor
FGFR1	Fibroblast growth factor receptor 1
FOX	Forkhead box (Fox gene family)
GAG	glycosaminoglycan
GEO	Gene Expression Omnibus
GFs	Growth factors
GO	Gene ontology
GPCCi001-A	Human induced pluripotent stem cell line created in Greater Poland Cancer Centre
hADSCs	Human adipose-derived stem cells
hiPSCs	Human induced pluripotent stem cells
HPGD	15-hydroxyprostaglandin dehydrogenase
HOX	Homeobox genes
IGF-1	Insulin-like growth factor 1
ITS	Insulin–transferrin–selenium
KEGG	Kyoto Encyclopedia Genes and Genomes
MMP-13	Matrix metallopeptidase 13
MSCs	Mesenchymal stem cells
PDGF-BB	Platelet-derived growth factor
RARB	Retinoic acid receptor, beta polypeptide
RMA	Robust Multiarray Average Normalization Algorithm
SCs	Stem cells
SEMA3C	Semaphorin 3C
SONFH	steroid-induced osteonecrosis of the femoral head
SOX	SRY (sex determining region Y)-box genes
TGF-β3	Transforming growth factor beta 3
VEGF	Vascular endothelial growth factor
VEGFR2	Vascular endothelial growth factor receptor 2
Wnt	Wingless-type MMTV integration site family
