# Supplementary material for: The Role of MicroRNAs in Early Chondrogenesis of Human Induced Pluripotent Stem Cells (hiPSCs)
Source: Int J Mol Sci. 2019 Sep 5;20(18):4371. doi: 10.3390/ijms20184371 (PMC6770352; doi:10.3390/ijms20184371)
Supplement: Supplementary file 1 [file ijms-20-04371-s001.zip › ijms-547545 suppl for final/capation.pdf]

Supplementary Figure 1. The chondrocyte-like cells differentiated from hiPSCs demonstrated characteristic of human chondrocytes such as the expression of the following markers: type II collagen, type IX collagen, aggrecan, COMP.

Supplementary Figure 2. The relationship between miRNAs engaged in the downregulation of the Wnt signaling pathway and genes regulated by them. ChiPS cells demonstrate decreased level of expression of markers characteristic for Wnt signaling pathway (red color) and upregulated level of expression of miRNAs that regulate those genes (green color). The intensity of used color of miRNAs and regulated genes were based on their FC values (ChiPS vs. hiPSCs). miRNAs regulate sets of genes at the same time.

Supplementary Table 1. The differentially expressed miRNAs with defined fold change and adj. p-values (hiPSCs vs. ChiPS).
