# Supplementary figures and images for: The Role of MicroRNAs in Early Chondrogenesis of Human Induced Pluripotent Stem Cells (hiPSCs)
Source: Int J Mol Sci. 2019 Sep 5;20(18):4371. doi: 10.3390/ijms20184371 (PMC6770352; doi:10.3390/ijms20184371)

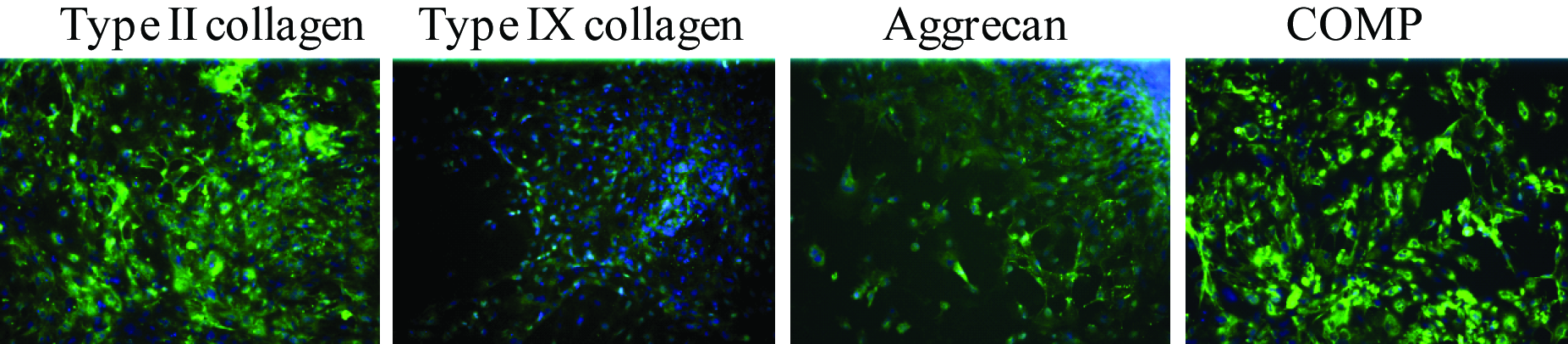

Supplement: Supplementary file 1 [file ijms-20-04371-s001.zip › ijms-547545 suppl for final/Supplement Fig. 1.tif]

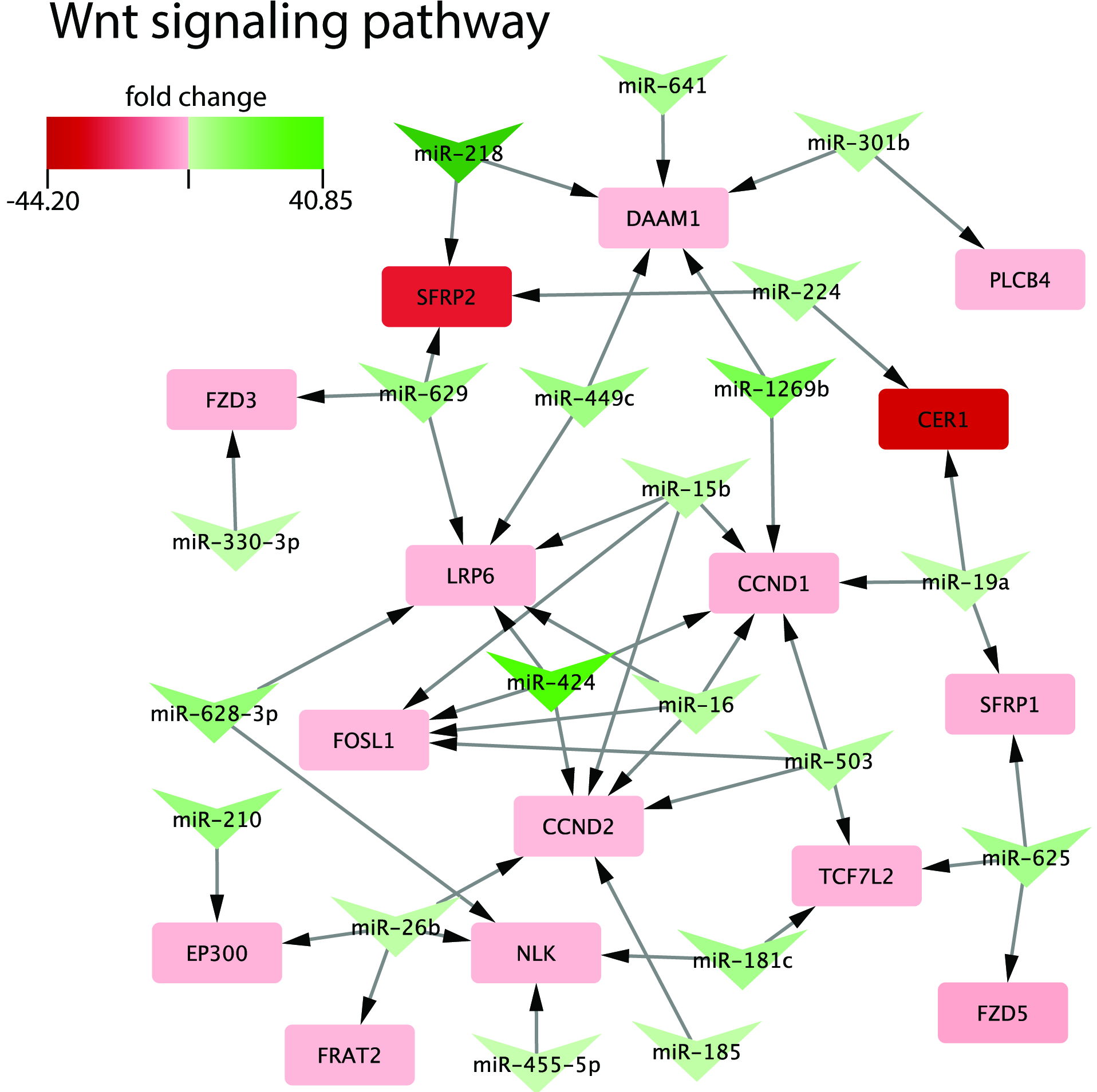

Supplement: Supplementary file 1 [file ijms-20-04371-s001.zip › ijms-547545 suppl for final/Supplement Fig. 2.tif]
